# Supplementary figures and images for: Oculomotor analysis to assess brain health: preliminary findings from a longitudinal study of multiple sclerosis using novel tablet-based eye-tracking software
Source: Front Neurol. 2023 Sep 6;14:1243594. doi: 10.3389/fneur.2023.1243594 (PMC10516298; doi:10.3389/fneur.2023.1243594)

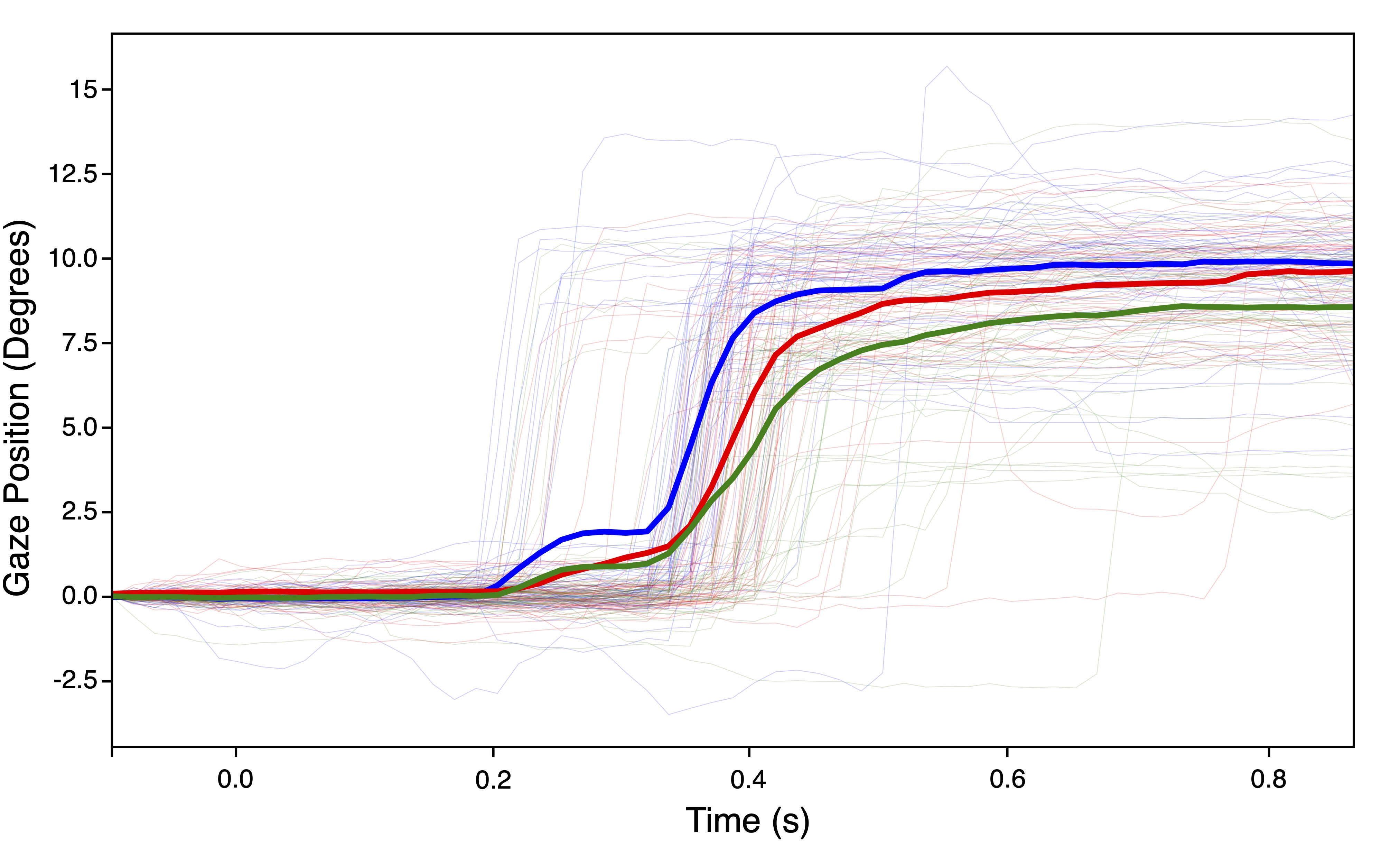

Supplement: Supplementary FIGURE 1 — Illustrated the individual trial gaze traces for each participant when performing large rightward saccades, color-coded based on the patient EDSS score (red: EDSS 0-2, blue: 2-4, green: 4-8), along with the average trace for each of the three EDSS groupings (n = 20). [file Image_1.JPEG]
